# Interobserver Variability in Ki-67 Index Assessment of Gastroenteropancreatic Neuroendocrine Neoplasms: A Comparative Evaluation of Three Counting Methods

**DOI:** 10.30699/ijp.2025.2055662.3423

**Published:** 2025-08-15

**Authors:** Emerine Selma Edwin, Pooja K Suresh, Hema Kini, Saraswathy Sreeram, Jyoti Kini, Sridevi HB, Sneha AR Rao

**Affiliations:** Department of Pathology, Kasturba Medical College Mangalore, Manipal Academy of Higher education, Manipal, India

**Keywords:** Neuroendocrine neoplasms; Gastroenteropancreatic neuroendocrine tumors; Ki-67; MIB-1; Visual estimation; ImageJ

## Abstract

**Background & Objective:**

Accurate evaluation of the Ki-67 proliferation index is critical in the grading and prognostication of neuroendocrine neoplasms (NENs). This study aimed to compare three different methods of Ki-67 index assessment in primary gastroenteropancreatic neuroendocrine neoplasms (GEP-NENs).

**Methods:**

The Ki-67 proliferation index was assessed by immunohistochemical (IHC) staining in 41 cases of primary GEP-NENs. Two pathologists independently evaluated the Ki-67 index using two visual estimation methods: eyeballing and eye-counting under a light microscope. A third method involved manual counting of Ki-67–positive and –negative nuclei on a captured image using ImageJ software. Interobserver and intermethod reproducibility were analyzed using Pearson’s correlation coefficient (R) and the intraclass correlation coefficient (ICC) to assess agreement among the four observers and between each observer and the manual method.

**Results:**

The ICC among the four observers was 0.993, indicating excellent interobserver agreement. The ICC between each observer’s score and the manual counting method was 0.994, also demonstrating high concordance.

**Conclusion:**

All three methods, eyeballing, eye-counting, and manual counting using ImageJ, proved to be comparably effective in assessing the Ki-67 proliferation index in GEP-NENs. Notably, the simpler microscopic techniques of eyeballing and eye-counting showed excellent agreement with the manual image-based method, supporting their reliability in routine practice.

## Introduction

Neuroendocrine neoplasms (NENs) arise from neuroendocrine cells distributed throughout the body, with the majority originating in the pulmonary and gastroenteropancreatic (GEP) systems (1). According to the 2019 World Health Organization (WHO) classification, gastroenteropancreatic NENs (GEP-NENs) are categorized into neuroendocrine tumors (NETs), neuroendocrine carcinomas (NECs), and mixed neuroendocrine–non-neuroendocrine neoplasms (MiNENs) based on tumor differentiation, mitotic index (MI), and Ki-67 proliferation index (2). Among these parameters, Ki-67 is a critical prognostic marker, and its accurate evaluation is essential for tumor grading and predicting patient outcomes (3,4).

Despite its importance, assessment of the Ki-67 index remains methodologically challenging. The most commonly used approach is eyeballing, which involves visual estimation of Ki-67 nuclear staining in the most proliferative ("hotspot") areas. However, this method suffers from significant interobserver variability, limited reproducibility, and is both labor-intensive and time-consuming (5). An alternative, though less commonly applied, method is manual image-based counting, where digital images of stained tumor sections are acquired at specific magnifications and the positively stained nuclei are manually quantified to calculate the labeling index. Advances in digital pathology have introduced software-based tools such as ImmunoRatio, QuPath, and ImageJ, which facilitate automated or semi-automated Ki-67 quantification (3,4,6). While digital image analysis (DIA) offers improved standardization, its widespread adoption is constrained by high costs, limited access in resource-limited settings, and the risk of inaccurate counts due to inclusion of non-neoplastic cells (5).

Given the critical role of Ki-67 in tumor grading and the ongoing need for reproducible and accessible assessment methods, this study aimed to evaluate and compare the reproducibility of Ki-67 index measurements using three approaches: eyeballing, eye-counting, and manual image-based counting using ImageJ, as performed by four independent observers. Our findings aim to contribute to the identification of a reliable and feasible method for Ki-67 evaluation in GEP-NENs.

## Materials and Methods

All histomorphologically confirmed cases of primary gastroenteropancreatic neuroendocrine neoplasms (GEP-NENs) received between January 2016 and September 2021 were included in this study. A total of 41 cases were evaluated, originating from the esophagus (1 case), stomach (6 cases), small intestine (16 cases), large intestine (15 cases), gallbladder (1 case), and pancreas (2 cases). Approval for this study was obtained from the Institutional Ethics Committee (IEC KMC MLR 12-2020/442). Formal written informed consent was waived, as the study utilized archival material.

Cases were excluded if tissue blocks and slides were unavailable or if the material was inadequate for immunohistochemistry (IHC).

### Immunohistochemistry

IHC for Ki-67 was performed in cases previously confirmed as NENs by positive staining for chromogranin A and/or synaptophysin. A monoclonal antibody against Ki-67 (Dako) was used following standard protocols, with breast carcinoma tissue serving as a positive control. Only nuclear staining in tumor cells was considered positive for Ki-67.

### Ki-67 Index Assessment

The Ki-67 labeling index was independently assessed by five reviewers (Observers 1–4 and a manual count) using three different methods. Tumor hotspots were identified using an Olympus CX21i microscope. The assessment methods were as follows:

**1. Eyeballing:**

Observers 1 and 3 estimated the percentage of Ki-67–positive tumor cells by visual scanning of the stained slides without performing individual cell counts. This qualitative method is commonly used in routine diagnostic settings (5).

**2. Eye-counting on microscope”**

Observers 2 and 4 performed real-time cell counting under a microscope. After identifying the tumor hotspot under low power, the field was switched to a high-power objective (40x), and a minimum of 500 tumor cells, including positively stained nuclei, were manually counted to calculate the Ki-67 index.

**3. Manual counting on a micrograph using freeware:**

**Fig 1:**
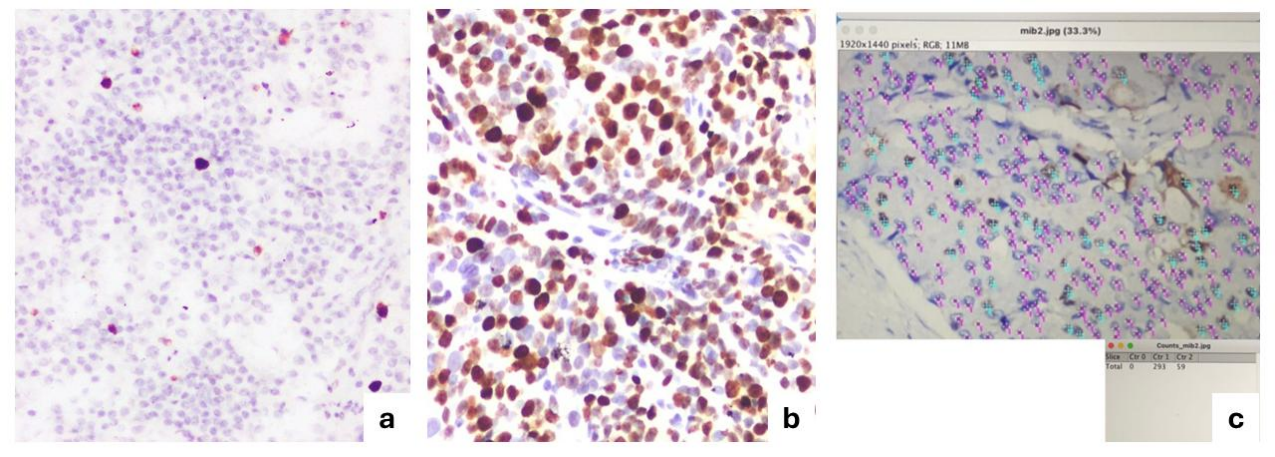
Microphotographic image of Ki67 with ImageJ pictiure.

For this method, representative hotspots were photographed at 10x magnification. The digital images were uploaded into ImageJ (a freeware image analysis tool), where positive and negative nuclei were manually marked and counted by two reviewers in consensus. (See Figure 1 for workflow.)

### Statistical Analysis

Reproducibility among the three counting methods was evaluated using Pearson’s correlation coefficient (R) to determine the pairwise correlation between the four observers, as well as between each observer and the manual image-based counting method.

a, b: Immunohistochemical image of Ki67 antibody showing low and high Ki67 index c: Ki-67 negative and positive tumour cells is highlighted purple and blue and counted in a freeware ImageJ.

## Results

We assessed interobserver variability in Ki-67 index estimation among pathologists with varying levels of experience using three different counting methods across 41 cases of GEP-NENs.

The intraclass correlation coefficient (ICC) among the four observers was 0.993, indicating excellent interobserver agreement. Similarly, the ICC between the observers’ scores and the manual image-based count was 0.994, again reflecting excellent concordance. These data are summarized in Table 1.

**Fig 2:**
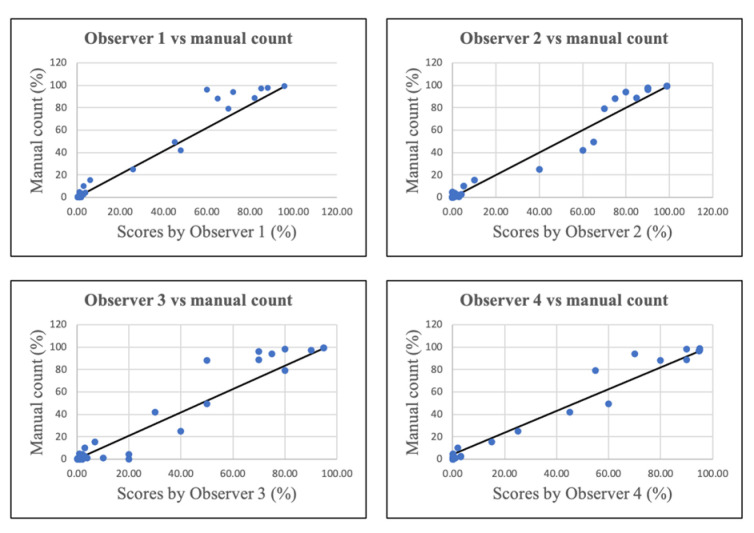
Correlation coefficients between eyeballing and eye-counting by observers and manual count on micrograph

Both eyeballing (Observers 1 and 3) and eye-counting under the microscope (Observers 2 and 4) demonstrated a high degree of correlation with the manual count performed on micrographs of tumor hotspots using ImageJ. The Pearson correlation coefficients between the different visual methods and the manual image-based method are graphically illustrated in Figure 2.

In each panel (A, Observer 1; B, Observer 2; C, Observer 3; D, Observer 4) the Ki-67 proliferation scores in percentage by eyeballing and eye-counting are plotted along the *x*-axis and the manual Ki-67 counts (%) on micrographs are plotted along the *y*-axis. A, Pearson r = 0.985; B, Pearson r = 0.986; C, Pearson r = 0.968; D, Pearson r = 0.988.

### Interobserver Correlation

Strong correlations were observed among all pairwise comparisons of the four observers, with Pearson correlation coefficients exceeding 0.95 in all instances. Specifically:

Observer 1 showed correlations of 0.98, 0.97, and 0.98 with Observers 2, 3, and 4, respectively.

Observer 2 demonstrated correlations of 0.98, 0.96, and 0.99 with Observers 1, 3, and 4, respectively.

Observer 3 showed correlations of 0.97, 0.96, and 0.95 with Observers 1, 2, and 4, respectively.

**Fig 3:**
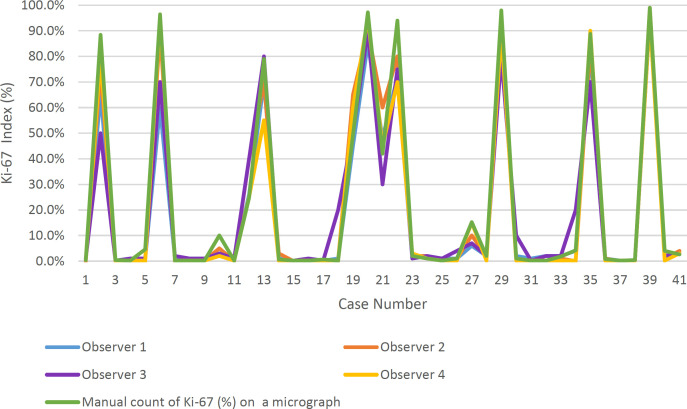
Ki-67 proliferation index values by all methods. Graphical representation of Ki-67 proliferation index values by all methods

These results are detailed in Table 2 and visualized in Figure 3.

**Table 2:** Correlation between each of the 4 observers’ scores represented by Pearson’s correlation coefficient r.

Variable 1	Variable 2			
	Observer 1	Observer 2	Observer 3	Observer 4
Observer 1	1	.987	.976	.980
Observer 2	.987	1	.969	.991
Observer 3	.976	.969	1	.957
Observer 4	.980	.991	.957	1

### WHO Tumor Grade Assignment

Tumor grades were assigned based on Ki-67 indices derived from each observer’s evaluation and the manual count, according to the 2019 WHO classification for GEP-NENs:

- Grade 1: Ki-67 <3%
- Grade 2: Ki-67 3%–20%
- Grade 3: Ki-67 >20%

The distribution of tumor grades based on the different methods and observers is presented in Table 3.

**Table 3:** Tumor grade according to WHO 2019 classification of GEP-NENs

Case no	Observer 1	Observer 2	Observer 3	Observer 4	Manual count on micrograph
1	G1	G1	G1	G1	G1
2	G3	G3	G3	G3	G3
3	G1	G1	G1	G1	G1
4	G1	G1	G1	G1	G1
5	G1	G1	G1	G1	G2
6	G3	G3	G3	G3	G3
7	G1	G1	G1	G1	G1
8	G1	G1	G1	G1	G1
9	G1	G1	G1	G1	G1
10	G2	G2	G2	G1	G2
11	G1	G1	G1	G1	G1
12	G3	G3	G3	G3	G3
13	G3	G3	G3	G3	G3
14	G1	G2	G1	G1	G1
15	G1	G1	G1	G1	G1
16	G1	G1	G1	G1	G1
17	G1	G1	G1	G1	G1
18	G1	G1	G2	G1	G1
19	G3	G3	G3	G3	G3
20	G3	G3	G3	G3	G3
21	G3	G3	G3	G3	G3
22	G3	G3	G3	G3	G3
23	G1	G1	G1	G2	G1
24	G1	G1	G1	G1	G1
25	G1	G1	G1	G1	G1
26	G1	G1	G2	G1	G1
27	G2	G2	G2	G2	G2
28	G1	G1	G1	G1	G1
29	G3	G3	G3	G3	G3
30	G1	G1	G2	G1	G1
31	G1	G1	G1	G1	G1
32	G1	G1	G1	G1	G1
33	G1	G1	G1	G1	G1
34	G2	G1	G2	G1	G2
35	G3	G3	G3	G3	G3
36	G1	G1	G1	G1	G1
37	G1	G1	G1	G1	G1
38	G1	G1	G1	G1	G1
39	G3	G3	G3	G3	G3
40	G1	G1	G1	G1	G2
41	G2	G2	G2	G2	G1

## Discussion

Ki-67 is a nuclear antigen expressed during active phases of the cell cycle, and its expression reflects the proliferative activity of tumor cells (4). The Ki-67 index has prognostic significance in various tumor types, including neuroendocrine neoplasms (NENs), breast carcinomas, and central nervous system tumors such as glioblastomas and oligodendrogliomas (2,6,7). In gastroenteropancreatic neuroendocrine neoplasms (GEP-NENs), the Ki-67 proliferation index is a critical parameter for tumor grading and staging, directly influencing clinical management. Its prognostic importance is evident from the substantial differences in overall survival observed between WHO grade 1 (G1) and grade 3 (G3) tumors (8).

Determining the Ki-67 index in NENs, however, remains a diagnostic challenge in surgical pathology due to the strict cutoff values defined by the WHO grading system. Over time, several counting approaches have been proposed (3–6,9). Among them, eyeballing remains the most widely adopted method in routine practice due to its simplicity and cost-effectiveness (10). The WHO recommends counting at least 500 tumor cells in the most proliferative ("hotspot") areas at scanning magnification, whereas the Royal College of Pathologists (RCP) advises counting at least 2,000 cells (2,11). Unfortunately, in practice, these thresholds are not always strictly adhered to, especially in resource-limited settings, raising concerns about poor reproducibility and interobserver variability, particularly in borderline cases near the G1/G2 or G2/G3 thresholds (3). This can lead to misclassification, either upgrading or downgrading tumors. Nonetheless, some studies have reported acceptable reliability with the eyeballing method. Our findings are consistent with those supporting its accuracy.

Several factors influence the accuracy of Ki-67 evaluation, including tumor heterogeneity, variability in marker expression, and hotspot selection (9). The reliability of visual estimation is also influenced by the pathologist’s experience, which can affect interobserver agreement (9). While senior pathologists may more reliably distinguish between high-grade and low-grade tumors, concerns persist regarding reproducibility. To explore this, our study included four practicing pathologists with experience ranging from 5 to 30 years. Despite this range, excellent concordance was observed among their assessments.

Reid et al. found manual counting on printed or photographed micrographs to be the most economical and reliable method in a comparative analysis of four counting techniques, eyeballing, automated digital image analysis (DIA), manual eye-counting under the microscope, and micrograph-based manual counting (5). Similarly, Young et al. reported that manual counting was more accurate than eyeballing; in their study, 37 of 93 cases were incorrectly graded when using the ENETS classification via eyeballing alone (12). Although micrograph-based manual counting is considered the gold standard, it is time-consuming and may require additional resources such as color printing.

Emerging methods using image analysis software offer automated Ki-67 quantification. These tools allow scanning of the entire slide, with hotspot regions selected by trained observers. The software then performs automated cell counting. While this reduces evaluation time, it introduces the risk of misidentifying non-tumor cells, such as endothelial, stromal, or inflammatory cells, as tumor cells, leading to inaccurate proliferation indices. The applicability of DIA also depends heavily on available infrastructure. While many pathology centers in high-income countries use DIA in routine practice, the associated costs, ranging from USD 50,000 to 150,000 for whole slide imaging (WSI) equipment, make them less accessible in low-resource settings. In our region, the cost of WSI devices is estimated at USD 50,000–70,000, underscoring the need for more accessible alternatives.

Most pathology laboratories, even in resource-constrained settings, have access to desktop computers or laptops. Thus, there is a strong rationale for developing simple, low-cost, and efficient software tools that can run on widely available devices. In our study, we used ImageJ, a free and open-source software, to manually quantify Ki-67, positive and –negative tumor cells on micrographs. While some studies have employed ImageJ with custom plug-ins and color-coded macros for automated detection, such approaches can introduce errors due to fragmented cells or false-positive staining (10). To mitigate these issues, we performed manual counting on static micrographs.

All three methods used in our study, eyeballing, eye-counting under the microscope, and micrograph-based manual counting, proved to be simple, economical, and reasonably accurate. Bologna-Molina et al. proposed a similar manual approach using digital images and a grid overlay to improve precision and reproducibility (13). Their method also limited the inclusion of proliferating non-neoplastic cells, a common source of error in computer-assisted analysis. Additionally, multiple observers can assess the same digital sample asynchronously, from different locations, making this method adaptable to remote or collaborative environments. However, despite these advantages, further validation of such techniques is still needed.

According to the College of American Pathologists (CAP), validation of an IHC assay requires a cohort of at least 20 cases, with ≥90% concordance between the new method and the gold standard (14). Our study included 41 cases, and demonstrated excellent agreement between observers (r = 0.957–0.991) and between observers and the manual count (r = 0.968–0.988), meeting CAP criteria for concordance.

In terms of time efficiency, the average time taken to assess one case was 2 minutes for eyeballing, 5 minutes for eye-counting, and 3 minutes for manual micrograph-based counting. Automated analyzers, while fast in execution, require an initial setup time (~10 minutes) to calibrate thresholds and exclude non-tumor elements, along with a certain level of technical training. Factors such as observer fatigue, variation in interpretation, object identification challenges, and image clarity can also impact the accuracy of visual estimation methods (10).

Interobserver variation in Ki-67 assessment has been widely studied. For instance, a recent study in breast cancer demonstrated a median variability of approximately 10% among observers evaluating 30 cases. In breast cancer, Ki-67 serves primarily as a prognostic biomarker and predictor of response to neoadjuvant chemotherapy (15). However, in GEP-NENs, Ki-67 evaluation plays a pivotal role not only in prognosis but also in diagnosis, grading, and management decisions. Thus, establishing reliable, reproducible, and accessible methods of assessment is essential.

## Conclusion

Accurate quantification of the Ki-67 proliferation index is essential for the proper grading and management of GEP-NENs. Our study demonstrates that visual estimation methods—eyeballing and eye-counting under the microscope—are comparable in accuracy to manual counting on digital micrographs. Moreover, the consistency of results among observers with different levels of experience highlights the robustness and clinical utility of these conventional methods. Given their simplicity, low cost, and reproducibility, these techniques remain valuable tools in routine pathology practice, particularly in resource-limited settings.

## Data Availability

There is no additional data separate from available in cited references.
